# Comparative Analysis of the Incidence of Selected Sexually Transmitted Bacterial Infections in Poland in 2010–2015: A Retrospective Cohort Study

**DOI:** 10.3390/jcm11040998

**Published:** 2022-02-14

**Authors:** Magda Orzechowska, Mateusz Cybulski, Elzbieta Krajewska-Kulak, Agnieszka Gniadek, Wiaczeslaw Niczyporuk

**Affiliations:** 1 Department of Epidemiology of Infectious Diseases and Supervision, National Institute of Public Health—National Research Institute, 00-791 Warsaw, Poland; 2 Department of Integrated Medical Care, Faculty of Health Sciences, Medical University of Bialystok, 15-096 Bialystok, Poland; mateusz.cybulski@umb.edu.pl (M.C.); elzbieta.krajewska-kulak@umb.edu.pl (E.K.-K.); 3 Institute of Nursing and Midwifery, Faculty of Health Sciences, Jagiellonian University Medical College, 31-501 Krakow, Poland; agnieszka.gniadek@uj.edu.pl; 4 Chair of Medical Sciences, Faculty of Health Sciences, Lomza State University of Applied Sciences, 18-400 Lomza, Poland; wniczyporuk@pwsip.edu.pl

**Keywords:** epidemiology, gonorrhoea, non-gonococcal urethritis, morbidity, sexually transmitted infections, syphilis, urogenital chlamydial infections, venereal diseases

## Abstract

Sexually transmitted infections are common infectious diseases. The main aim of this study was to perform a comparative analysis of the incidence of bacterial sexually transmitted infections in 2010–2015 in Poland, taking into account the administrative division of the country into provinces. This was a retrospective study. The analysed data came from the Centre for Health Information Systems of the Ministry of Health and constituted information being the epidemiological surveillance system in Poland. The analysis included data on the incidence of primary and secondary syphilis, gonorrhoea and non-gonococcal urethritis and genital infections. The overall incidence rates were disproportionately lower than European rates and those presented in studies from other countries. Young people, between 20 and 29 years of age, were the key groups at the highest risk of infection. The incidence rate of primary and secondary syphilis was lower in Poland than in Europe or America, but some regions, such as Mazovia and Lodz provinces, were found to have a higher incidence rate than other European rates. The reported incidence of gonorrhoea in Poland was also significantly lower compared with other countries, with a significantly higher number of infections in males than in females, and this was also one of the highest rates in EU countries. During the study period, the number of non-gonococcal genital infections systematically decreased, while in other countries of the European region, the incidence was among the highest of all sexually transmitted infections.

## 1. Introduction

Sexually transmitted infections (STIs) are clinical syndromes caused by pathogens that can be acquired and transmitted through sexual activity. STI pathogens are mainly bacteria, viruses, fungi and protozoa. The total number of sexually transmitted pathogens currently exceeds 35, with each pathogen likely to have multiple subtypes that may produce different clinical manifestations [1].

Sexually transmitted infections are the most common infectious diseases worldwide, with over 357 million new cases each year in the 15–49 age group [2]. This group of infections is responsible for far-reaching health, social and economic consequences. Failure to diagnose and treat sexually transmitted diseases at an early stage can result in serious complications and consequences [3]. Worldwide, the prevalence of bacterial sexually transmitted infections (STIs) has shown a significant upward trend in recent years. This trend is primarily reflected in the increased incidence of syphilis in many countries, e.g., Germany [4]. According to World Health Organization (WHO) estimates from 2012, approximately 6 million new syphilis infections occur annually worldwide [5]. In 2015, the European Centre for Disease Prevention and Control (ECDC) recorded 28,000 infections [6], corresponding to an increase of 49% compared to 2010. Gonorrhoea is now the fourth most common sexually transmitted infection worldwide (70.3 million cases per year) after cervicella, chlamydial infections and genital warts [7]. In 2015, the ECDC received almost 70,000 notifications [6]. Genitourinary chlamydial infections (131 million worldwide) were the second most common infection in 2012 after *Trichomonas vaginalis* infection [5]. *Mycoplasma genitalium* is considered to be the second most common causative agent of non-gonococcal urethritis after *Chlamydia trachomatis* [8].

The aim of this study was to perform a comparative analysis of the incidence of sexually transmitted infections between 2010 and 2015 in Poland. The specific objectives were:

1. To compare epidemiological data on selected bacterial sexually transmitted infections, such as syphilis, gonorrhoea and non-gonococcal urethral and genital infections (NGU), by year, taking into account socio-demographic data (i.e., age, sex) and the structure of incidence for all forms;
2. Comparison of the number of cases and the incidence rate in the subsequent years.

The following research hypotheses were set for the main objective and specific objectives:

1. The number of bacterial sexually transmitted infections in Poland was on the rise between 2010 and 2015;
2. The highest incidence of all STIs was shown in the age group 25–44 years;
3. Sexually transmitted infections are more commonly diagnosed in men.

## 2. Materials and Methods

### 2.1. Research Materials and Methods

This was a retrospective study. For this purpose, a detailed comparative analysis of the incidence rates of bacterial sexually transmitted infections from 2010 to 2015 was performed, taking into account socio-demographic data such as age and gender. Data on the incidence of primary and secondary syphilis, gonorrhoea and non-gonococcal urethritis and genital infections were analysed.

The analysed data came from the Centre for Health Information Systems of the Ministry of Health and from provincial offices. The sources of analysed data were collective annual reports on patients treated in the dermatology and venereology outpatient clinic MZ-14 from 16 voivodeships submitted to voivodeship offices competent for the place of providing health services. The available data concerned the number of cases with a division by gender of patients taking into account 9 age groups.

Due to the nature of the study, which involved an analysis of epidemiological data, the approval of the local Bioethics Committee was not required.

### 2.2. Statistical Analysis

Data were processed using: Microsoft Excel 2020 (Microsoft Corporation, Albuquerque, NM, USA) and Statistica 13.0 (StatSoft Company, Hamburg, Germany).

The obtained data on the incidence of syphilis, gonorrhoea and non-gonococcal urethritis and genital infections were assessed for the frequency of occurrence among women and men. Although the analysis was nationwide, regional variation by provinces was also taken into account.

The analysis covered the entire period of 2010–2015. For each disease, statistics were determined for each province to show the differences in incidence among men and women per 100,000 inhabitants. Since the number of women and men was similar, it was possible to perform a comparative analysis of the number of cases.

The assessment of the statistical significance of differences between the incidence in age groups included data from the entire study period, i.e., 2010–2015, excluding groups <15 years of age, where the small number of cases did not allow the use of statistical inference techniques.

Using a logistic regression model, the statistical significance of differences between morbidity in the compared age groups was assessed. Morbidity assessments in each age group were made while controlling for the year of onset, and the values obtained represented the mean annual incidence in 2011–2015.

Odds ratio (OR) and risk ratio (RR) values were taken at a confidence interval (CI) of 95%. The test probability was *p* = 0.05 (CI 95%).

## 3. Results

### 3.1. Characteristics of the Studied Population

The characteristics of the studied population are presented in Table 1.

### 3.2. Incidence of Selected Bacterial Sexually Transmitted Infections between 2010 and 2015

#### 3.2.1. Primary and Secondary Syphilis

The spatial distribution of syphilis incidence rates for provinces in the analysed time period was stable. In most of the analysed periods, higher incidence rates were observed in the provinces of central Poland (i.e., Mazovia and Lodz provinces), while the lowest rate occurred in south-eastern Poland (i.e., Świętokrzyskie, Subcarpathian and Podlasie provinces).

Between 2010 and 2015, 6669 patients were treated for syphilis in dermatology and venereology outpatient clinics in Poland regardless of the stage of the disease. The analysed data take into account the forms of syphilis by the duration of infection and symptomology. Men accounted for the vast majority of patients (5100, 76.5%), with 1569 women (23.5%) affected during this period.

In the analysed period, early acquired syphilis (<1-year duration) was diagnosed in 4633 cases, accounting for 69.5% of the total number of syphilis cases. These cases are of particular epidemiological importance as they reflect the actual dynamics of the incidence due to the duration of the infection. Primary syphilis accounted for 18.5% of treated cases, secondary early syphilis for 16%, secondary recurrent syphilis for 14.5%, and early latent syphilis for 20.5%. Late clinical stages, i.e., developing > 1 year after infection, and indeterminate stages accounted for nearly ^1^/_3_ incidence, i.e., 28.1%. Between 2010 and 2015, 157 cases of congenital syphilis were reported, accounting for 2.4% of all forms.

The incidence of syphilis over the years under study, regardless of the stage of infection, showed an upward trend, i.e., from 2.5/100,000 in 2010 to 3.3/100,000 in 2015, with the highest value recorded in 2013. Considering the administrative division by provinces, the highest number of cases were recorded in Mazovia Province—2017 (31.0%), Lodz Province—827 (12.4%) and Silesia Province—652 (9.8%). Single cases were reported in Subcarpathian and Świętokrzyskie provinces—46 each over 6 years. A higher incidence of syphilis was also associated with a higher urbanisation index in the region (R = 0.51).

#### 3.2.2. Gonorrhoea

There was an increase in the number of gonorrhoeal infections in Poland between 2010 and 2015, which almost doubled in 2015 compared to the year at the beginning of the study, i.e., from 301 cases in 2010 to 500 in 2015. The highest number of cases was recorded in 2012 (733). During this period, 2876 patients were treated for gonorrhoea in dermatology and venereology outpatient clinics nationwide, including 2485 men (86.4%) and 391 women (13.6%).

Nearly 50.0% of *Neisseria gonorrhoeae* infections were diagnosed in the Mazovian Province and 25.0% in the Pomeranian Province. The highest incidence rates in the analysed period were recorded in the Pomeranian Province in 2012–2014, i.e., 14.3/100,000, 7.1/100,000 and 5/100,000 inhabitants, respectively, and in 2015, the Mazovian Province (5.6/100,000).

The largest steady increase in the incidence of gonorrhoea occurred in the Mazovia Province, i.e., from 2.6/100,000 in 2010 to 5.6/100,000 in 2015.

The incidence rate of gonorrhoea decreased between 2000 and 2015 in Lubusz, Podlasie, Lodz, Świętokrzyskie and West Pomeranian provinces. Although there was no statistically significant correlation between the frequency of reporting gonorrhoea and the urbanisation index (R = 0.39), a certain upward tendency in the incidence with a higher urbanisation index of the province was observed.

#### 3.2.3. Non-Gonococcal Urethritis and Genital Inflammation

Between 2010 and 2015, the MZ-14 reports indicated 2213 patients treated for non-gonococcal urethritis (NGU), including 1620 men (73%) and 593 women (27%). Compared to 2010, the number of non-gonococcal urethral infections decreased by ^1^/_3_ in 2015. High variability in the number of reported cases of NGU during the analysed period was noted.

The incidence rate of NGU showed a decreasing trend in 2014 and 2015, although it was also very low in some provinces in 2011. It is difficult to identify regions with significantly higher incidence, although it was certainly lower in south-eastern Poland in recent years.

Additionally, in the case of non-gonococcal genital infections (R = 0.44), a certain tendency for the incidence to increase with the higher urbanisation rate of the province was noted.

The numerical characteristics of the distribution of descriptive statistics of the incidence of the analysed sexually transmitted infections in the following years are presented in Table 2.

### 3.3. Assessment of the Direction of Change in Incidence in Individual Provinces

The Spearman rank correlation coefficient was used to assess systematic changes in incidence rates in individual provinces. The obtained results for individual provinces and diseases are presented in Table 3.

A systematic decrease in the incidence or lack of correlation with provinces was demonstrated for all analysed diseases in Lublin, Świętokrzyskie and West Pomeranian provinces.

The analysis showed that:

In Lower Silesia, a rather marked increase in the incidence of gonorrhoea and moderate upward trends were observed for syphilis and NGU, with only the incidence of gonorrhoea showing statistical significance;

A systematic decrease in the incidence of syphilis was observed in Lesser Poland during the analysed period (R = −0.94);

A statistically significant strong upward trend in the incidence rate of gonorrhoea was noted in Greater Poland (R = 0.89).

### 3.4. Correlations between Incidence Rates and the Individual Diseases

A correlation analysis was performed between the mean incidence rates between 2010 and 2015 and the diseases analysed (Table 4).

The analysis of the results showed that:

There is a correlation between the incidence rates of individual diseases, but there is no relationship between them;

The weakest correlation was for gonorrhoea;

The diseases for which the incidence rates were most clearly correlated were: non-urethral urethritis and syphilis.

### 3.5. Incidence by Age Group

Table 5 shows the incidence rates of the analysed bacterial sexually transmitted infections in individual years and age groups. The level of incidence of primary and secondary syphilis was significantly higher in the age group 20–44 years compared to the other groups. The highest incidence was among persons aged 25–29 years. For gonorrhoea, similarly to syphilis, the rates were by far the highest in the 20–44 age group, with those aged 20–29 years and particularly 25–29 years being particularly vulnerable groups. Similarly, in the case of non-gonococcal urethritis and genital infections, the highest incidence was among those aged 20–29 years. Variability in incidence across years was also noted.

The odds ratio values for contracting the analysed bacterial sexually transmitted infections in a given age group are presented in Table 6.

The large variation in the incidence of primary and secondary syphilis by age resulted in statistically significant OR values for each age group. The estimated risk of acquiring syphilis was strongly associated with age. It was the highest in persons aged 25–29 years and was six times higher than in those aged 15–19 years or 45–64 years. A sharp (i.e., five-fold) increase in the risk of infection after the age of 20 years (OR = 3.17) should be noted. At the same time, the risk of acquiring syphilis decreased significantly in persons over 45 years of age.

The large variation in the incidence of gonorrhoea by age resulted in a statistically significant OR value for each age group relative to the whole population. As in the case of syphilis, a strong relationship was observed for the risk of gonorrhoea by age. The highest risk was observed in persons aged 25–29 years; it was six times higher than in those aged 15–19 years or 45–64 years. There was also a sharp (i.e., five-fold) increase in the risk of infection after the age of 20 years (OR = 3.02). At the same time, the risk of acquiring gonorrhoea decreased significantly in persons over 45 years of age.

The large variation in the incidence of non-gonococcal urethritis in relation to age resulted in the statistical significance of OR values for almost every age group. The exception was the 15–19 years group, for which the incidence level did not differ from that for the total population. Invariably, as in the case of the sexually transmitted infections discussed above, the highest risk of infection was for young people, with at least a four-fold increase in risk after 20 years of age, followed by a decrease after the age of 30 years.

### 3.6. Incidence by Gender

Women accounted for less than half of patients with syphilis and gonorrhoea across provinces. The lowest proportion of women was reported for gonorrhoea (9.2%) (Table 7).

The incidence of primary and secondary syphilis was definitely higher among men—the incidence rates in the provinces were even a dozen times higher among men than among women. In Mazovia Province, the incidence rates were 57.4/100,000 inhabitants for men and less than 5/100,000 inhabitants for women, respectively. In Podlasie and Świętokrzyskie, the incidence rates of syphilis among women and men were similar, i.e., men constituted more than half of the diagnosed cases, 54.1 and 58.3%, respectively. The proportion of women was much lower and, at the same time, the lowest in the Mazovian province (less than 9%), which means that women were affected more than 10 times less often than men. The analysis has shown that only in the Podlasie and Świętokrzyskie provinces, there was no statistically significant difference in the incidence of syphilis between men and women (Table 8). In the remaining regions, men fell ill significantly more often. The incidence rate of syphilis among men was more than 11 times higher than among women (RR = 11.51, CI 95%).

Equally pronounced differences were found for the incidence of gonorrhoea, which was higher in almost all provinces in the male group (Table 8). The percentage of affected men exceeded 80% (the lowest percentage in Warmia-Masuria, Lubusz and Subcarpathian Province). This means that men were at least four times more likely to be affected with gonorrhoea than women; in most provinces, the proportion was much higher. The analysis showed that in all provinces, there were statistically significant differences in the incidence of gonorrhoea among men and women. The incidence rates in some regions were even several times higher among men than among women. In general, however, a somewhat more cautious assessment of gender differences in the incidence should be made since, as is readily apparent, the accuracy of RR estimates was not high due to the relatively small number of gonorrhoea cases, especially among women. Nevertheless, even considering the lower ends of the RR confidence intervals, the incidence is more than 10 times higher, especially among men, in regions such as Mazovia and Pomerania.

In almost all provinces, the incidence of NGU was several times higher in the male group. In Mazovia in 2010–2015, the incidence rate was nearly 17 per 100,000 men, while among women, it was more than three times lower (Table 8). In two provinces, i.e., Subcarpathian and Opole Province, no cases were found among women during the period under study. The share of men in the total number of cases was highly varied—from the complete absence of cases to a lower number of cases than in women in Lower Silesian Province (ca. 44%). In most regions, with the exception of Lower Silesian and Lublin Province, there was a statistically significant difference between the incidence among women and men. In all these regions, a higher incidence was noted among men.

## 4. Discussion

### 4.1. Syphilis

Data on the incidence of primary and secondary syphilis and late (tertiary) syphilis available in Poland showed an upward trend, with the highest number of patients in 2013. At the same time, it should be mentioned that a steady increase was recorded for late syphilis. During this period, the incidence in Europe was estimated at 4.1/100,000 in 2010 to 6.5/100,000 in 2016. In the United States, the incidence of primary and secondary syphilis was 6.3/100,000 in 2014. There was a 15.1% increase in incidence compared to 2013 and 40% compared to 2010 [9]. The average incidence in Poland was lower, ranging from 2.5/100,000 in 2010 to 3.5/100,000 in 2013 and 3.3/100,000 in 2015.

The incidence of syphilis in Germany was 8.5/100,000 in 2015 and 7.1/100,000 in 2014. The highest incidence was reported in Berlin (31/100,000) and Hamburg (19.7/100,000). The lowest incidence was reported in Schleswig-Holstein, Brandenburg and Thuringia. Incidence rates were particularly high in several large cities, such as Leipzig (21.8/100,000) and Cologne (31.9/100,000) [10]. In Mecklenburg, which borders Poland, there was a 154.2% increase in incidence between 2013 and 2014. The reason for the increase was the trend of increasing syphilis diagnosis in small- and medium-sized towns. The incidence of syphilis in Hansestadt increased by 703% between 2013 and 2014 [10].

In 2015, the syphilis prevalence in Saxony was 8.6 cases/100,000 inhabitants. The incidence rate was 16/100,000 among males and 1.5/100,000 among females. Male predominance was also present in the national reports. The proportion of women among syphilis patients in Germany in 2015 was 6.2%. The incidence of syphilis was 16.2/100,000 population in men and 1/100,000 population in women and generally matched the values in Saxony [11].

In Europe, the prevalence of syphilis among males compared to females (male-to-female ratio, MF) has been increasing over the last decade, where the ratio was 2.5 in 2005, 6.2 in 2014 and 8.0 in 2015. Significant differences were observed across countries. The male-to-female ratio for syphilis is above 10 in France, Germany, Ireland, the Netherlands, Slovenia, Norway and the United Kingdom, while it is below 2 in Cyprus, Finland, Bulgaria, Latvia, Lithuania, Romania and Slovakia [12]. Although data on the sex of syphilis patients are available in Polish reporting, it is not reported to the ECDC (only the summed number of cases of both sexes). The MF indicator reflects the most frequent circumstances of transmission, i.e., the nature of sexual contact. As the proportion of men among syphilis patients increases, infections are more common as a result of homosexual contact.

### 4.2. Gonorrhoea

The global estimate of gonorrhoea incidence among women was 0.8% (0.6–1.0%), with values ranging from 0.3 to 1.7%, depending on the world region [7]. Gonorrhoea is included in European surveillance (European Sexually Transmitted Infections Surveillance Network). National data on gonorrhoea are sent to the European Centre for Disease Prevention and Control in Stockholm by 24 countries with comprehensive and mandatory reporting of gonorrhoea cases and 4 countries with sentinel surveillance (Belgium, the Netherlands, France and Hungary) [13].

In countries where reporting diagnosed cases is mandatory, the incidence rate was 19/100,000, with the highest rates in the UK (i.e., 67/100,000), Denmark (i.e., 49/100,000) and Ireland (i.e., 28/100,000). The lowest rates, i.e., below 1/100,000, were observed in Croatia, Cyprus and Romania [13]. During the analysed period, the incidence in Poland in particular years was very low and also oscillated around the value of 1/100,000. In 2015, the number of cases was 214% of the number of cases in 2010 in Europe (more than doubled). In Poland, the same difference was lower at 166%, but it was noted that a record number of cases were reported in 2012, and the increase was 243% compared to 2010. Between 2008 and 2017, there was a six-fold increase in *N. gonorrhoeae* infections in France and Portugal and more than three times as many confirmed cases in Denmark and Ireland. It should be noted that some of the reported increases over time may be due to improved national surveillance systems and the use of more sensitive tests [14]. Data analysis indicated a decrease in recorded gonorrhoea cases in Poland since 2012.

European data on age came from 25 countries, including Poland, but in heterogeneous forms and were not included in the analysis (i.e., 7% of all cases). The highest proportion of reported cases was in people aged 25–34 years (36%) and 15–24 years (35%). Incidence rates for each age group in 2015 were highest among 20–24-year-olds (100/100,000 population), while in Poland, the rate described in the 20–29-year-old age group was much lower (8.4/100,000). In persons aged 15–19 years, the incidence was significantly higher among women (i.e., 62/100,000) compared to men (i.e., 41/100,000), while in older age groups, the rates were higher among men. The highest age and sex ratios were reported for men aged 20–24 years (137/100,000) [13]. Similar incidence trends were reported in Poland, but the incidence values were several times lower. In all analysed years, the highest incidence rates were found among men aged 25–29 years and ranged from 9.4 to 14.6/100,000; similar values were reported for men aged 30–39 years. In Poland, as in Europe, the incidence of the disease decreased with age. The incidence was significantly lower in the female group irrespective of age and had incidence values below 1, except for in 2012, when it was over 3/100,000 among women between 20 and 29 years of age. The male-to-female ratio from ECDC data indicated a 3.6-fold higher incidence among men (32/100,000 men and 8.8/100,000 women) in 2015. The results obtained for Poland indicated a significantly higher incidence in male representatives (between 10 and 13 times). At the same time, this was one of the highest rates in EU countries, after Croatia (17) and Slovenia (14) [13]. The increasing trend in the incidence in men indicates an urgent need to implement preventive measures in this group.

In 2015, the number of reported cases of gonorrhoea in Europe increased by 144% compared to 2008. The increase was seen in both sexes, but it was more pronounced among men (+158%) compared to women (+76%) [13]. The increase in the number of cases in Poland was more dynamic than the European one and was 66% during the analysis period, followed by 143% and 82% in 2012 and 2013, respectively. Data on risk group membership of STI patients are not routinely available in Poland. This information should be included in individual incidence reports in the surveillance carried out by the National Sanitary Inspectorate.

In the US, gonorrhoea incidence was highest in 2015 at 124/100,000 population, with a 13% increase compared to 2014. Women aged 15 to 24 years and men aged 20 to 24 years were predominantly affected [15].

Due to the lack of mandatory reporting, there are no current data on the epidemiology of gonorrhoea in Germany. Until 2001, *N. gonorrhoeae* infections were reportable in Germany. Current data on the prevalence of gonorrhoea in Saxony are available due to the extended STI reporting regulation (IfSG, Meldeverordnung entsprechend dem Infektionsschutzgesetz). In Saxony, there has been a striking increase in reported *N. gonorrhoeae* infections since 2009. In 2015, the prevalence of reported gonorrhoea cases in Saxony was 20.4/100,000, compared to 17.9/100,000 in 2014. In terms of incidence values, the rate was broadly in line with data from the European Centre for Disease Prevention and Control (ECDC), which had an average incidence of 20/100,000 in 27 European countries in 2014. It seems interesting to compare the main cities in Saxony, which showed that Leipzig had the highest incidence in 2015 (43.8/100,000). The highest incidence was followed by Dresden (29.6/100,000) and Chemnitz (41.7/100,000). The reasons for such a high incidence in Leipzig are related to more frequent laboratory diagnoses by physicians and particularly good reporting of laboratory practices, but the incidence of risky behaviour in the study was only an assumption [16]. These are the largest cities in the region, which lies on the border with Poland. The association of the prevalence of gonorrhoea with the level of urbanisation was also confirmed in an incidence analysis in Portugal, where the relative risk of gonorrhoea in association with the urbanisation index was estimated at 1.65 [17]. The data from Poland available in the study concerned only provinces.

### 4.3. Non-Gonococcal Urethritis and Genital Infections

Using data from 2005–2012, the prevalence of chlamydia in women worldwide was determined to be 4.2% (95% UI: 3.7–4.7%), with values ranging regionally between 1.8 and 7.6%, with the highest in the Americas and Western Pacific region. Chlamydia infections were significantly higher than those estimated for syphilis and gonorrhoea (0.5 and 0.8%). The prevalence of chlamydia in men worldwide was 2.7% (95% UI: 2.0–3.6%), with regional values ranging from 1.3 to 5.2%. For cysticercosis, the global estimate was 5.0% (4.0–6.4%), with regional values ranging from 1.0 to 11.5% [7].

The prevalence of chlamydia among women, as defined by the World Bank under one of the set targets, ranged from 2.4 to 6.9% compared to 1.6 to 4.2% among men, respectively. The global incidence rate for chlamydia was estimated to be 38 per 1000 in women (by region: 15–72) and 33 per 1000 in men (by region: 13–64). *T. vaginalis* infection was globally almost 12 times more common among women than men and 10 times more common when considering the European Region. The prevalence of chlamydia and cysticercosis infections increased proportionally in the regions as economic levels declined, so the Global Burden of Disease estimates are very close to those of the WHO [18,19]. Uncertainty was greatest for the prevalence estimates of cysticercosis. In all regions, the estimated prevalence of chlamydia in men and women and cervicella in women was higher than the prevalence of gonorrhoea or syphilis.

However, it was not possible to compare global incidence trends, as WHO’s cyclical estimates were characterised by variable methods of analysis. In addition, fewer surveys of STI prevalence among representative samples of the general population are being conducted and published over time, making it increasingly challenging to have sufficient data to generate reliable estimates.

In Germany, precise data are not available due to the lack of routine reporting, but the Saxony region is covered. In 2015, in Saxony, the incidence was around 100/100,000, which has been consistent since 2009. The infected were mainly men, three times more often compared to women. About 75% of female infections were diagnosed at screening tests available in Germany since 2009 in this group [20].

The data available in Poland and used for the analysis concerned the incidence of non-gonococcal urethritis and genital infections without differentiating the aetiological agent. During the period analysed, the number of reported infections decreased by 33%. As in Saxony, infections were significantly more frequent in men, which also coincided with World Bank estimates. The maximum incidence values in Poland oscillated around 4/100,000, with some regions having zero incidence. This difference should also be interpreted in the context of symptomatic infections, while the reporting data from the Saxony region come from screening and mostly report asymptomatic infections. The reported values in the different years were highly variable between regions. In Germany, the incidence was most common among older people, as the same incidence values were reported for the 20–29 and 30–39 age groups. In Poland, a survey conducted among school and university students in large cities allows concluding that the incidence of genital chlamydial infections is similar to the global level (1.1–6.6% depending on the city) [21].

The average prevalence of chlamydial infections in Europe (26 countries) in 2014 was 187/100,000 population. Again, there are significant differences in reported cases between countries. In 2014, 83% of all chlamydial infections in Europe were reported from Denmark (549/100,000), Norway (486/100,000), Sweden (375/100,000) and England (366/100,000). The numerical discrepancies in reported chlamydial infections can be attributed to, among other things, differences in testing strategies, diagnosis, surveillance strategies (e.g., screening programmes) and the extent to which cases in different countries in Europe go unreported. In 2014, the prevalence in the United States was 479/100,000 population [22,23,24].

*Mycoplasma genitalium* infections are considered the second most common cause of non-gonococcal sexually transmitted bacterial infections in Western Europe, after *C. trachomatis*. The recently published European guidelines for *M. genitalium* infections assume that the microorganism causes urethritis unrelated to chlamydia and gonococci in 10–35% of men and women [25,26,27].

Epidemiological studies in the UK have confirmed that *M. genitalium* is a pathogen significantly frequently responsible for genitourinary infections. The prevalence of *M. genitalium* was 1.2% in men and 1.3% in women in 2015, with infections in 16–19-year-olds generally undetectable, while the proportion diagnosed in 25–34-year-olds was 2.1%. It is noteworthy that 94.4% of men and more than half of women (56.2%) with *M. genitalium* showed no symptoms of STIs [8].

## 5. Conclusions

The incidence of all forms of syphilis and gonorrhoea increased statistically significantly during the study period, while the incidence of non-gonococcal genital infections decreased. The highest incidence of all analysed sexually transmitted infections in Poland was observed in the age group of 20–29 years. Higher incidence rates among persons aged 40–49 were noted for syphilis. Men were significantly more frequently diagnosed with gonorrhoea and syphilis.

The worsening epidemic situation with respect to sexually transmitted infections, the inefficiency of the current surveillance system and reduced funding for their diagnosis and prevention, combined with inadequate legal solutions, make it necessary to undertake new legal and organisational strategies aimed at improving the reproductive health in Poland in terms of sexually transmitted infections.

## Figures and Tables

**Table 1:** Characteristics of the studied population.

Characteristics		Syphilis	Gonorrhoea	Non-Gonococcal Urethritis and Genital Infections	Total
Overall number of patients *n* (%)		6669 (56.7%)	2876 (24.5%)	2213 (18.8%)	11,758 (100%)
Sex *n* (%)	Men	5100 (76.5%)	2485 (86.4%)	1620 (73.2%)	9205 (78.3%)
	Women	1569 (23.5%)	391 (13.6%)	593 (26.8%)	2553 (21.7%)
M/F ratio		3.25	6.36	2.73	3.61
Age range * n (%)	<1	10 (0.2%)	1 (0.0%)	0 (0.0%)	11 (0.1%)
	1–9	13 (0.2%)	1 (0.0%)	6 (0.4%)	20 (0.2%)
	10–14	8 (0.1%)	0 (0.0%)	2 (0.1%)	10 (0.1%)
	15–19	148 (2.7%)	73 (2.8%)	75 (4.4%)	296 (3.0%)
	20–24	862 (15.3%)	453 (17.5%)	329 (19.7%)	1644 (16.6%)
	25–29	1217 (21.7%)	621 (24.0%)	466 (27.9%)	2304 (23.3%)
	30–44	2314 (41.2%)	1059 (40.9%)	538 (32.2%)	3911 (39.6%)
	45–64	890 (15.8%)	338 (13.1%)	225 (13.4%)	1453 (14.7%)
	≥65	159 (2.8%)	44 (1.7%)	32 (1.9%)	235 (2.4%)

*—age ranges cover 2011–2015; in 2010, this data was not recorded.

**Table 2:** Distribution of descriptive statistics of incidence per 100,000 inhabitants for the analysed sexually transmitted infections between 2010 and 2015.

	Year	M	Me	*SD*	Min.	Max.
Primary and secondary syphilis	2010	1.43	1.28	0.88	0.14	3.24
	2011	1.39	1.17	1.14	0.08	3.95
	2012	1.40	1.29	1.16	0.19	4.60
	2013	2.14	1.83	2.00	0.09	7.72
	2014	1.68	1.22	1.52	0.28	5.25
	2015	1.84	1.63	1.74	0.24	7.66
Gonorrhoea	2010	0.69	0.42	0.70	0.07	2.56
	2011	0.57	0.23	0.86	0.04	3.44
	2012	1.65	0.36	3.56	0.09	14.28
	2013	1.17	0.40	1.95	0.00	7.14
	2014	1.00	0.39	1.57	0.05	5.00
	2015	0.91	0.36	1.53	0.12	5.57
Non-gonococcal urethritis and genital infection	2010	1.35	1.04	1.28	0.00	4.01
	2011	0.91	0.59	1.00	0.04	3.35
	2012	0.92	0.68	0.97	0.09	3.60
	2013	1.16	0.58	1.17	0.00	3.62
	2014	0.83	0.34	1.08	0.00	3.43
	2015	0.89	0.31	1.16	0.00	3.93

Abbreviations: M—mean, Max.—maximum, Me—median, Min.—minimum, SD—standard deviation.

**Table 3:** Spearman rank correlation coefficients (R) of STI occurrence for provinces.

Province	Sexually Transmitted Infections		
	Primary and Secondary Syphilis	Gonorrhoea	NGU
Lower Silesia	0.66	0.83 *	0.60
Kuyavian-Pomeranian	0.31	−0.43	−0.54
Lublin Province	−0.14	0.09	−0.60
Lubusz Province	0.77	−0.60	0.49
Lodz Province	0.03	−0.37	0.09
Lesser Poland	−0.94 *	0.66	−0.20
Mazovia Province	0.94 *	0.94 *	−0.20
Opole Province	0.54	0.71	−0.15
Subcarpathian Province	0.54	−0.03	−0.78
Podlasie Province	−0.37	−0.49	−0.31
Pomeranian	0.83 *	0.37	−0.31
Silesia	−0.20	−0.54	−0.66
Świętokrzyskie Province	0.37	−0.60	−0.77
Warmia-Masuria Province	0.71	0.83 *	−0.03
Greater Poland	0.49	0.89 *	0.54
West Pomeranian Province	−0.54	−0.26	−0.71

*—statistically significant correlations (*p* ≤ 0.05).

**Table 4:** Correlations between incidence rates of specific sexually transmitted infections in Poland between 2010 and 2015.

Incidence Rates in Years 2010–2015	Primary and Secondary Syphilis	Gonorrhoea	NGU
Primary and secondary syphilis	1	0.44	0.66
*p*	-	0.0872	0.0052 *
Gonorrhoea	0.44	1	0.57
*p*	0.0872	-	0.0202 *
NGU	0.66	0.57	1
*p*	0.0052 *	0.0202 *	-

*—statistically significant result (*p* ≤ 0.05).

**Table 5:** Incidence of selected bacterial sexually transmitted infections in age groups by year per 100,000 population.

Sexually Transmitted Infection	Age Group(years)	Incidence Rate per 100,000 Persons				
		2011	2012	2013	2014	2015
Primary and secondary syphilis	<1	0.00	0.53	0.83	0.27	0.55
	1–9	0.00	0.00	0.00	0.00	0.14
	10–14	0.00	0.00	0.00	0.00	0.06
	15–19	1.04	0.86	1.41	0.93	1.21
	20–24	4.58	3.78	6.40	5.15	6.51
	25–29	5.62	5.61	7.43	5.12	7.45
	30–44	2.57	3.27	4.66	4.22	4.04
	45–64	0.92	0.84	1.33	0.97	0.93
	≥65	0.15	0.13	0.25	0.12	0.15
Gonorrhoea	<1	0.26	0.00	0.00	0.00	0.00
	1–9	0.00	0.00	0.00	0.03	0.00
	10–14	0.00	0.00	0.00	0.00	0.00
	15–19	0.60	0.99	0.52	0.64	0.66
	20–24	1.80	4.12	4.37	2.85	4.40
	25–29	1.98	5.48	4.73	3.86	4.48
	30–44	1.37	3.78	2.32	2.42	2.18
	45–64	0.61	0.80	0.65	0.66	0.49
	≥65	0.04	0.31	0.14	0.17	0.12
Non-gonococcal urethritis and genital infections	<1	0.00	0.00	0.00	0.00	0.00
	1–9	0.00	0.00	0.06	0.00	0.11
	10–14	0.00	0.00	0.00	0.00	0.11
	15–19	0.43	0.27	1.08	0.59	1.21
	20–24	2.85	2.71	2.64	2.14	2.24
	25–29	2.65	2.76	4.47	2.18	3.28
	30–44	1.01	1.30	1.21	1.04	1.56
	45–64	0.51	0.30	0.57	0.38	0.38
	≥65	0.09	0.07	0.18	0.12	0.10

**Table 6:** Odds ratio of incidence of selected bacterial sexually transmitted infections by age group.

Sexually Transmitted Infection	Age Group (years)	Disease Risk Assessment		
		OR	OR (95% CI)	*p*
Primary and secondary syphilis	15–19	0.66	0.56–0.77	<0.001 *
	20–24	3.17	2.90–3.46	<0.001 *
	25–29	3.76	3.47–4.07	<0.001 *
	30–44	2.26	2.10–2.43	<0.001 *
	45–64	0.60	0.55–0.66	<0.001 *
	≥65	0.09	0.07–0.12	<0.001 *
Gonorrhoea	15–19	0.59	0.49–0.73	<0.001 *
	20–24	3.02	2.73–3.35	<0.001 *
	25–29	3.55	3.23–3.90	<0.001 *
	30–44	2.09	1.92–2.28	<0.001 *
	45–64	0.56	0.50–0.62	<0.001 *
	≥65	0.13	0.10–0.17	<0.001 *
Non-gonococcal urethritis and genital infections	15–19	0.85	0.70–1.04	0.1103
	20–24	3.06	2.72–3.44	<0.001 *
	25–29	3.71	3.33–4.12	<0.001 *
	30–44	1.48	1.33–1.64	<0.001 *
	45–64	0.52	0.45–0.59	<0.001 *
	≥65	0.14	0.10–0.18	<0.001 *

* statistically significant result (*p* ≤ 0.05).

**Table 7:** Summary incidence for the assessed diseases in the female group.

Disease	% of Women in the Total Number of Cases in 2010–2015				
	M	Me	*SD*	Min.	Max.
NGU	23.0%	24.0%	15.3%	0.0%	56.4%
Syphilis	23.7%	23.0%	10.4%	8.6%	45.9%
Gonorrhoea	9.2%	7.4%	5.4%	0.0%	19.5%

**Table 8:** Comparison of incidence of selected bacterial sexually transmitted infections by sex between 2010 and 2015.

Sexually Transmitted Disease	Province	Women		Men		M/F RR (95% CI)	*p*
		*n*	*I*	*n*	*I*		
Primary and secondary syphilis	Lower Silesia	29	1.92	215	15.35	8.00 (5.43–11.79)	<0.001 *
	Kuyavian-Pomeranian	67	6.21	193	19.02	3.06 (2.32–4.04)	<0.001 *
	Lublin Province	42	3.77	92	8.79	2.33 (1.62–3.35)	<0.001 *
	Lubusz Province	32	6.11	78	15.68	2.57 (1.70–3.87)	<0.001 *
	Lodz Province	175	13.28	417	34.74	2.61 (2.19–3.12)	<0.001 *
	Lesser Poland	53	3.07	265	16.27	5.30 (3.95–7.12)	<0.001 *
	Mazovia Province	138	4.98	1459	57.43	11.51 (9.67–13.71)	<0.001 *
	Opole Province	15	2.88	58	11.91	4.13 (2.34–7.29)	<0.001 *
	Subcarpathian Province	5	0.46	18	1.73	3.75 (1.39–10.11)	0.0049 *
	Podlasie Province	17	2.77	20	3.43	1.23 (0.65–2.36)	0.5231
	Pomeranian	18	1.53	90	8.05	5.25 (3.17–8.71)	<0.001 *
	Silesia	135	5.67	376	16.91	2.98 (2.45–3.63)	<0.001 *
	Świętokrzyskie Province	10	1.54	14	2.26	1.47 (0.65–3.30)	0.3525
	Warmia-Masuria Province	34	4.60	106	14.95	3.25 (2.21–4.78)	<0.001 *
	Greater Poland	51	2.87	246	14.60	5.09 (3.77–6.88)	<0.001 *
	West Pomeranian Province	20	2.27	145	17.32	7.62 (4.78–12.17)	<0.001 *
Gonorrhoea	Lower Silesia	3	0.20	26	1.86	9.35 (2.83–30.89)	<0.001 *
	Kuyavian-Pomeranian	25	2.32	159	15.67	6.76 (4.44–10.31)	<0.001 *
	Lublin Province	2	0.18	30	2.87	15.94 (3.81–66.68)	<0.001 *
	Lubusz Province	9	1.72	45	9.05	5.27 (2.57–10.77)	<0.001 *
	Lodz Province	12	0.91	68	5.66	6.22 (3.37–11.49)	<0.001 *
	Lesser Poland	4	0.23	53	3.25	14.05 (5.09–38.82)	<0.001 *
	Mazovia Province	102	3.68	1272	50.07	13.58 (11.10–16.61)	<0.001 *
	Opole Province ^#)^	0	0.00	14	2.87	30.98 (1.85–519.41)	0.0001 *
	Subcarpathian Province	2	0.18	11	1.06	5.74 (1.27–25.88)	0.0101 *
	Podlasie Province	3	0.49	38	6.51	13.29 (4.10–43.04)	<0.001 *
	Pomeranian	51	4.34	668	59.77	13.76 (10.35–18.29)	<0.001 *
	Silesia	9	0.38	101	4.54	12.01 (6.07–23.76)	<0.001 *
	Świętokrzyskie Province	1	0.15	34	5.48	35.61 (4.87–260.11)	<0.001 *
	Warmia-Masuria Province	8	1.08	33	4.65	4.29 (1.98–9.30)	0.0001 *
	Greater Poland	3	0.17	52	3.09	18.30 (5.72–58.60)	<0.001 *
	West Pomeranian Province	2	0.23	36	4.30	18.93 (4.56–78.62)	<0.001 *
Non-gonococcal urethritis and genital infections	Lower Silesia	62	4.10	48	3.43	0.84 (0.57–1.22)	0.3482
	Kuyavian-Pomeranian	63	5.84	145	14.29	2.45 (1.82–3.29)	<0.001 *
	Lublin Province	35	3.15	41	3.92	1.24 (0.79–1.95)	0.3409
	Lubusz Province	25	4.77	176	35.38	7.41 (4.88–11.27)	<0.001 *
	Lodz Province	111	8.42	197	16.41	1.95 (1.54–2.46)	<0.001 *
	Lesser Poland	15	0.87	96	5.89	6.79 (3.94–11.69)	<0.001 *
	Mazovia Province	153	5.53	426	16.77	3.03 (2.52–3.65)	<0.001 *
	Opole Province ^#)^	0	0.00	8	1.64	18.16 (1.05–314.69)	0.0035 *
	Subcarpathian Province ^#)^	0	0.00	40	3.84	84.48 (5.19–1373.87)	<0.001 *
	Podlasie Province	12	1.96	38	6.51	3.32 (1.74–6.36)	0.0001 *
	Pomeranian	21	1.79	53	4.74	2.65 (1.60–4.39)	0.0001 *
	Silesia	17	0.71	78	3.51	4.91 (2.91–8.30)	<0.001 *
	Świętokrzyskie Province	1	0.15	17	2.74	17.80 (2.37–133.78)	0. 0001 *
	Warmia-Masuria Province	34	4.60	96	13.54	2.94 (1.99–4.35)	<0.001 *
	Greater Poland	8	0.45	30	1.78	3.96 (1.82–8.64)	0.0002 *
	West Pomeranian Province	40	4.54	127	15.17	3.34 (2.34–4.76)	<0.001 *

*n—*number of cases in 2010–2015, *I—*incidence per 100,000 inhabitants, *RR—*relative risk of male versus female incidence, *p—*assessment of statistical significance of differences in incidence between men and women, ^#)^ RR estimated by means of a specific adjustment on a numerical value equal to 0, *—statistically significant result (*p* ≤ 0.05).

## Data Availability

Data are available upon reasonable request.
